# Epidemiology of Bone Fracture in Female Trauma Patients Based on Risks of Osteoporosis Assessed using the Osteoporosis Self-Assessment Tool for Asians Score

**DOI:** 10.3390/ijerph14111380

**Published:** 2017-11-13

**Authors:** Cheng-Shyuan Rau, Shao-Chun Wu, Pao-Jen Kuo, Yi-Chun Chen, Peng-Chen Chien, Hsiao-Yun Hsieh, Ching-Hua Hsieh

**Affiliations:** 1 Department of Neurosurgery, Kaohsiung Chang Gung Memorial Hospital, Kaohsiung 83301, Taiwan; ersh2127@cloud.cgmh.org.tw; 2 Department of Anesthesiology, Kaohsiung Chang Gung Memorial Hospital, Kaohsiung 83301, Taiwan; shaochunwu@gmail.com; 3 Department of Plastic Surgery, Kaohsiung Chang Gung Memorial Hospital, Kaohsiung 83301, Taiwan; bow110470@gmail.com (P.-J.K.); libe320@yahoo.com.tw (Y.-C.C.); venu_chien@hotmail.com (P.-C.C.); sylvia19870714@hotmail.com (H.-Y.H.)

**Keywords:** female, osteoporosis, Osteoporosis Self-Assessment Tool for Asians (OSTA), trauma, propensity-score matching, femoral fracture, bone mineral density (BMD)

## Abstract

*Background*: Osteoporotic fractures are defined as low-impact fractures resulting from low-level trauma. However, the exclusion of high-level trauma fractures may result in underestimation of the contribution of osteoporosis to fractures. In this study, we aimed to investigate the fracture patterns of female trauma patients with various risks of osteoporosis based on the Osteoporosis Self-Assessment Tool for Asians (OSTA) score. *Methods*: According to the data retrieved from the Trauma Registry System of a Level I trauma center between 1 January 2009 and 31 December 2015, a total of 6707 patients aged ≥40 years and hospitalized for the treatment of traumatic bone fracture were categorized as high-risk (OSTA < −4, *n* = 1585), medium-risk (−1 ≥ OSTA ≥ −4, *n* = 1985), and low-risk (OSTA > −1, *n* = 3137) patients. Two-sided Pearson’s, chi-squared, or Fisher’s exact tests were used to compare categorical data. Unpaired Student’s *t*-test and Mann–Whitney *U*-test were used to analyze normally and non-normally distributed continuous data, respectively. Propensity-score matching in a 1:1 ratio was performed with injury mechanisms as adjusted variables to evaluate the effects of OSTA-related grouping on the fracture patterns. *Results*: High- and medium-risk patients were significantly older, had higher incidences of comorbidity, and were more frequently injured from a fall and bicycle accident than low-risk patients did. Compared to low-risk patients, high- and medium-risk patients had a higher injury severity and mortality. In the propensity-score matched population, the incidence of fractures was only different in the extremity regions between high- and low-risk patients as well as between medium- and low-risk patients. The incidences of femoral fractures were significantly higher in high-risk (odds ratio [OR], 3.4; 95% confidence interval [CI], 2.73–4.24; *p* < 0.001) and medium-risk patients (OR, 1.4; 95% CI, 1.24–1.54; *p* < 0.001) than in low-risk patients. In addition, high-risk patients had significantly lower odds of humeral, radial, patellar, and tibial fractures; however, such lower odds were not found in medium- risk than low-risk patients. *Conclusions*: The fracture patterns of female trauma patients with high- and medium-risk osteoporosis were different from that of low-risk patients exclusively in the extremity region.

## 1. Background

Osteoporosis is characterized by the deterioration of bone mass and microarchitecture, leading to impaired bone strength and subsequently fragility fracture [1]. Osteoporotic fractures are defined as low-impact fractures resulting from low-level trauma, such as a fall from a standing height or less that would not ordinarily result in fracture [2,3]. However, osteoporotic fracture is not straightforwardly defined, which sometimes causes misunderstanding. For example, although large studies have shown that nearly all types of fractures occur more often in patients with low bone mineral density (BMD) irrespective of the site [4,5], a low BMD alone might not fully detect the risk of osteoporotic fractures [4] and fractures are not always associated with low BMD [6]. In addition, under such definition, bone fragility does not presumably contribute to fractures associated with a high-level trauma. In a study that compared the BMD of a random sample of women who sustained fractures in either low- or high-level trauma events, the results revealed that, in a high-energy trauma, patients with osteoporosis are more prone to fracture than those without osteoporosis [7]. The exclusion of high-level trauma fractures may result in the underestimation of the contribution of osteoporosis to fractures [7].

The BMD measured at the lumbar spine and hip is currently the standard assessment tool in diagnosing osteoporosis. The relationship between low BMD and major osteoporotic fractures, including the spine [8,9], hip [10,11], humerus [12,13], and forearm [14], has already been established. Considering that advanced age and low body weight are strongly associated with low BMD and increased risk of bony fracture [6,15,16], the World Health Organization (WHO) developed the Osteoporosis Self-Assessment Tool for Asians (OSTA) score calculated using the following formula: (body weight (kg) − age (year)) × 0.2 to identify women at risk for osteoporosis [17]. A significant positive correlation was found between the OSTA index and T-scores of BMD measured by dual energy X-ray absorptiometry at the femoral neck [18,19]. In this developmental study, OSTA performed better than other osteoporotic indices by showing a sensitivity of 91%, specificity of 45%, and receiver operating characteristic curve of 0.79 at the cutoff of −1 [17]. In addition, at the cutoff of −1, the difference in OSTA performance was minimal regardless of using the femoral neck and lumbar spine BMD as reference [20]. Based on the OSTA scores, patients could be stratified as with low (OSTA > −1), medium (−1 ≥ OSTA ≥ −4), and high risk (OSTA < −4) for sustaining osteoporosis [21,22]. It is estimated that the probability of a patient with an OSTA score >−4 not having osteoporosis is 99.3% [23]. According to OSTA score, the risk of osteoporosis is 61%, 15% and 3% for those patients with high-, medium- and low-risk osteoporosis, respectively [17]. With strong correlations for the populations in Taiwan [21,24], China [20], Korea [25], Singapore [26], Malaysia [23], Thailand [27], and Philippines [28], OSTA has been validated as an effective and feasible screening tool to identify patients at risk for sustaining osteoporosis [17,18,23,26,28,29,30,31].

In the emergency department (ED), the terms of fracture modifiers such as “low trauma” and “fragility” are challenging both for the patients and providers. The forces applied to a bone are hardly defined merely from a description of the event. In addition, quantifying the level of applied skeletal force is almost impossible in clinical practice. Over-consideration of this context may be misleading the physician in managing the patients. Currently, little is known regarding the impact of osteoporosis on the fracture patterns of patients with trauma. The main question is, in the event of trauma, is the location of fractures in patients at high- or medium risk for osteoporosis tend to be different from those at low-risk for osteoporosis? Therefore, this study aimed to investigate the fracture patterns of patients with different risks of osteoporosis based on the OSTA score of female trauma patients in a Level I trauma center.

## 2. Methods

### 2.1. Study Design

OSTA was calculated using the following formula: (body weight (kg) − age (year)) × 0.2 [17]. Female patients aged ≥40 years and hospitalized for the treatment of all-cause trauma were included in the study. Patients with incomplete OSTA values were excluded. Patients were stratified as with low (OSTA > −1), medium (−1 ≥ OSTA ≥ −4), and high risk (OSTA < −4) of sustaining osteoporosis. Among the total 23,705 patients enrolled in the Trauma Registry System from 1 January 2009 to 31 December 2015, 6707 met the inclusion criteria. Of them, 1585 were high-risk, 1985 were medium-risk, and 3137 were low-risk patients. Detailed patient information was retrieved, including age; weight (kg); height (cm); comorbidities such as diabetes mellitus (DM), hypertension (HTN), coronary artery disease (CAD), congestive heart failure (CHF), and cerebrovascular accident (CVA); trauma mechanism; initial Glasgow Coma Scale (GCS) score upon arrival at the ED; severity score of Abbreviated Injury Scale (AIS) in each body region; Injury Severity Score (ISS); rates of associated bone fractures; length of stay (LOS) at the hospital; rate of admission in the intensive care unit (ICU); and in-hospital mortality [32,33]. According to the definition of body mass index (BMI) by the World Health Organization [34,35], the patients were deemed as obese (BMI ≥ 30 kg/m^2^), overweight (BMI of <30 but ≥25 kg/m^2^), normal-weight (BMI of <25 but ≥18.5 kg/m^2^), and underweight (BMI of <18.5 kg/m^2^) and normal-weight patients with a. The GCS has been used as a triage tool since 1974 to assess the severity of neurologic deficits and predict the prognosis in patients with conscious disturbance [36,37,38] by focusing on the important functions of the central nervous system, consisting of eye-opening (score 1–4), verbal (score 1–5), and motor responses (score 1–6), and accordingly categorizes the patients into severe (GCS score, 3–8), moderate (GCS score, 9–12), or mild (GCS score, 13–15) groups of patients with disturbed consciousness [39]. The AIS is an internationally accepted anatomy-based measurement of injury severity with a simple numeric method for ranking specific injuries in an individual [40]. The AIS assess the severity of the anatomical injury representing with minor injury (1), moderate injury (2), serious to critical (3–5), and maximal injury (6), which indicates the survival status of the patient. To summarize a single patient’s multiple injures into a single score, the ISS was created based on the AIS severity values, that is, the summation of the squares of the severity digit in the AIS of the most severe injuries, in three of six predefined body regions [41]. The ISS is expressed as the median and interquartile range (IQR, Q1–Q3). Data collected were compared using the IBM SPSS Statistics for Windows version 20.0 (IBM Corp., Armonk, NY, USA). Categorical data were compared using chi-squared test or two-sided Fisher’s exact test were used. Levene’s test was used to estimate the homogeneity of variance of continuous data, then ANOVA (GCS, ISS, LOS and height) and ANCOVA (age and weight), with Games-Howell post-hoc test, were performed to assess the differences of high-risk or medium-risk vs. low-risk of patients. The continuous data were expressed as mean ± standard deviation. Odds ratios (ORs) of the associated conditions and bone fractures of the patients were calculated with 95% confidence intervals (CIs). To minimize the confounding effects of non-randomized assignment in assessing the mortality outcome among different OSTA groups, propensity scores were calculated using NCSS software (NCSS 10, NCSS Statistical Software, Kaysville, UT, USA) using the following covariates: age, comorbidities, and trauma mechanisms. A 1:1 matched set of comparable study populations for high- vs. low-risk and medium- vs. low-risk patients was created using the greedy method after adjusting the trauma mechanisms which act as confounding factors. Binary logistic regression was performed to assess the effects of OSTA-related grouping on the mortality outcomes. *p*-values < 0.05 were considered statistically significant.

### 2.2. Ethical Statement

The institutional review board (IRB) of Chang Gung Memorial Hospital approved this study before its implementation, with an approval number 201600518B0 & 201600352B0. Informed consents were waived according to IRB regulations.

## 3. Results

### 3.1. Injury Characteristics and Severity of the Patients

**Figure 1:**
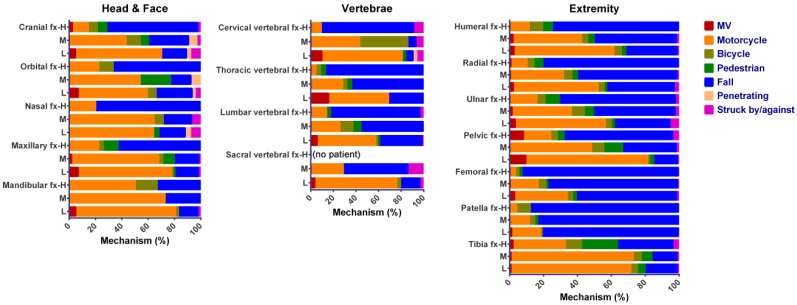
Composition of high-, medium-, and low-risk patients according to OSTA score in various injury mechanisms.

Table 1 shows that the mean age of high- and medium-risk patients was significantly higher than that of low-risk patients. The mean body weight of high- and medium-risk patients was significantly lower than that of low-risk patients. Both high- and medium-risk patients had higher odds of DM, HTN, CAD, and CVA than low-risk patients did (Table 2). In addition, high-risk, but not medium-risk, patients had higher odds of CHF than low-risk patients did. Regarding the injury mechanisms, high-risk as well as medium-risk and low-risk patients were remarkably different (Figure 1). Compared to low-risk patients, injuries due to fall and bicycle accidents were higher in high- and medium-risk patients, but fewer patients sustained injuries from a motor vehicle or motorcycle accident, a penetration injury, and being struck by/against objects. The percentage of high-risk patients sustaining a fall accident is much higher (80.6%) than that of medium-risk (51.0%) and low-risk patients (30.6%). In addition, pedestrian-related injuries were significantly higher in high-risk, but not medium-risk patients, than that of low-risk patients (Table 2).

As fewer patients had a GCS score of ≥13 and more patients had a GCS score of 9–12, GCS scores were significantly lower in high-risk patients than in low-risk patients (Table 2), notwithstanding that the GCS score difference between these groups was less than one point, whereas GCS scores were insignificantly different between medium- and low-risk patients (Table 1). The AIS analysis, under the criteria of AIS ≥3, revealed that high- and medium-risk patients sustained significantly higher rates of extremity injuries and lower rates of abdominal injuries than low-risk patients had. In addition, high-risk patients sustained significantly higher rates of head/neck injuries but lower rates of thoracic injuries than low-risk patients had. Compared to low-risk patients, a significantly higher ISS was found in high- and medium-risk patients. When stratified by ISS (<16, 16–24, or ≥25), more high-risk patients had an ISS of 16–24 and fewer had an ISS of <16 than low-risk patients did. In contrast, the percentage of patients with an ISS of <16 or 16–24 in medium-risk patients was insignificantly different than low-risk patients had. In patients with an ISS of ≥25, the percentage of high-risk or medium-risk patients was insignificantly different than low-risk patients had.

### 3.2. Outcomes and Associated Fractures of the Patients

High- and medium-risk patients had 3.6-fold (95% CI, 2.17–5.83; *p* < 0.001) and 2.2-fold (95% CI, 1.33–3.74; *p* = 0.002) higher odds of mortality than low-risk patients had, respectively (Table 2). High-risk patients had a higher proportion of patients admitted to the ICU (19.4% vs. 12.3%, respectively; *p* < 0.001) but not a significant difference of hospital LOS than low-risk patients did. However, the hospital LOS and proportion of patients admitted to the ICU were insignificantly different between medium- and low-risk patients.

Regarding the associated fractures (Table 3), high- and medium-risk patients had an 8.3-fold (95% CI, 7.13–9.56; *p* < 0.001) and 2.8-fold (95% CI, 2.45–3.28; *p* < 0.001) odds of femoral fracture than low-risk patients had, respectively. On the other hand, compared to low-risk patients, high-risk patients had a lower odds of facial, humeral, radial, pelvic, patella, and tibial fractures; and medium-risk patients had lower odds of cranial, facial, and tibial fractures.

### 3.3. Associated Fractures of the Propensity-Score Matched Patients

The injury mechanisms varied widely among the patients with different levels of risk. To minimize the confounding effects of injury mechanisms on the incidences of associated fracture, a separate set of propensity-score matched comparable study populations for high- and medium-risk vs. low-risk patients, respectively, was created for comparison. After propensity score matching, the outcome was compared in the 1268 well-balanced pairs of high- and low-risk patients and 1932 medium- and low-risk patients (Table 4). In these pairs of propensity-score matched patients, the injury mechanisms were insignificantly different between high- and low-risk patients (Supplementary Materials Table S1) and between medium- and low-risk patients (Supplementary Materials Table S2). The incidence of fractures was only different in the extremity regions between high- and low-risk as well as medium- and low-risk patients. The propensity-score matched between high- and low-risk patients revealed a 3.4-fold (95% CI, 2.73–4.24; *p* < 0.001) odds of femoral fracture in high-risk patients compared to low-risk patients. In addition, high-risk patients had significantly lower odds of humeral (OR, 0.7; 95% CI, 0.50–1.00; *p* = 0.047), radial (OR, 0.5; 95% CI, 0.39–0.71; *p* < 0.001), patellar (OR, 0.5; 95% CI, 0.27–0.78; *p* = 0.004) and tibial (OR, 0.6; 95% CI, 0.38–0.83; *p* = 0.004) fractures than low-risk patients had. The propensity-score matched between medium- and low-risk patients revealed that a 1.4-fold (95% CI, 1.24–1.54; *p* < 0.001) odds of femoral fracture was observed in medium-risk patients than low-risk patients had. No other significant difference on the incidence of fractures was found between medium- and low-risk patients.

### 3.4. Associated Extremity Fractures of the Patients in Fall Accidents

The patients sustained a fall injury had been further analyzed to identify the associated fractures of extremities. As expected, the mean age and weight were significantly higher but height was significantly lower in the high- or medium-risk patients than that of low-risk patients (Table 5). There were also more underweight and normal-weight but less overweight and obese patients of high- and medium-risk patients than those of low-risk patients (Table 6). In fall accidents, high- and medium-risk patients had a 4.9-fold (95% CI, 4.03–5.87; *p* < 0.001) and 2.3-fold (95% CI, 1.87–2.76; *p* < 0.001) odds of femoral fracture than low-risk patients had, respectively. On the other hand, compared to low-risk patients, high-risk patients had lower odds of humeral, radial, pelvic, patella, and tibial fractures; and medium-risk patients had a lower odds of patella and tibial fractures.

## 4. Discussion

This study compared the clinical fracture patterns of hospitalized female trauma patients, irrespective of injury mechanisms, based on the OSTA classification of associated risk to osteoporosis. In this study, high- and medium-risk patients were significantly older, had higher incidences of comorbidity, and were more frequently injured from a fall and bicycle accident than low-risk patients had. Compared to low-risk patients, high- and medium-risk patients had a higher injury severity and mortality. High-risk, but not medium-risk, patients had a higher proportion of patients admitted to the ICU than low-risk patients had. However, there was no significant difference of hospital LOS between high-risk or medium-risk patients and low-risk patients. After the attenuation of confounding effects to the mechanism of injury in the propensity-score matching, the incidence of fractures was only different in the extremity region between high- or medium-risk patients and low-risk patients. The incidences of femoral fractures are significantly higher in high-risk (OR 3.4; 95% CI 2.73–4.24; *p* < 0.001) and medium-risk (OR 1.4; 95% CI 1.24–1.54; *p* < 0.001) patients than low-risk patients had. In addition, high-risk patients had significantly lower odds of humeral, radial, patellar, and tibial fracture than low-risk patients had; however, such lower odds was not found between medium-risk and low-risk patients.

The existing literature suggests that OSTA can suitably be used in screening patients with increased risk of osteoporosis. As OSTA only considers two risk factors, i.e., age and body weight, the fracture pattern is not surprisingly deeply influenced by these two risk factors. With significantly older age in high- and medium-risk patients, higher incidences of comorbidity and injuries from a fall and bicycle accident were encountered more frequently than low-risk patients had. In this study, the percentage of high-risk patients sustaining a fall accident was much higher (80.6%) than that of medium-risk (51.0%) and low-risk (30.6%) patients. In a fall accident, the force impacts directly onto the posterolateral aspect of the greater trochanter, making the femoral neck particularly vulnerable to fractures [42]. In older age, the proximal femoral fractures occur not necessarily with high energy [43]. Obviously, the injury mechanisms may have a great impact on the fracture patterns found in patients with different OSTA scores. Therefore, comparison of propensity-score matched populations was performed in this study to reduce the confounding effect of injury mechanisms and the results indicated the incidences of fractures was different mainly in the extremity region between the patients with different risk of osteoporosis.

Regarding the body weight, obesity was once thought to protect people from having a fracture because of the observation that the bone in obese people was less osteoporotic. In a fall, the soft tissue padding may protect obese people against pelvic and hip fractures. In addition, the wrist was also protected from an impact because obese people tend to fall backward or sideways rather than forward, and together with impaired protective reactions to falling [44]. However, several conditions associated with obesity have adverse effects on bone health through various mechanisms, such as reduced physical activity, co-medications, and decreased 25-hydroxyvitamin D levels and consequent increased serum levels of parathyroid hormones [44]. Obese patients (body mass index of >30) had been reported to have an increased fracture severity and were more likely to suffer a complex injury resulting from a fall from a standing position [45]. The Global Longitudinal study of Osteoporosis in Women (GLOW) showed that obese women had higher incidences of ankle and upper leg fractures [46]. Obesity is increasingly associated with increased risk of fracture, albeit the pathogenesis of fracture in obese individuals have not yet been clearly defined [15]. The effect of obesity on fracture risk is site-dependent, the risk being increased for some fractures (humerus, ankle, and upper arm) and decreased for others (hip, pelvis, and wrist) [15]. Furthermore, the relationship between obesity and fracture may also vary by age and ethnicity [15]. Notably, obesity is not equal to the body weight, which used to calculate OSTA. Therefore, results of obesity researches should be cautiously interpreted.

Common osteoporotic fracture sites include bones that bear weight (such as the spine, pelvis, and hip) or bones that take most of the stress in a fall from a standing height or less (such as upper arm, forearm, and wrist) [6]. Notably, in this study that included patients with all trauma causes, not all fractures are due to osteoporosis. However, the forces applied to a bone during the accident is hard to define in clinical practice. The force impact on the bone may greatly vary even with the same mechanism. In the study conducted by Sanders et al. who revealed that excluding high trauma fractures may underestimate the prevalence of bone fragility fractures, 77.1% (835/1084) and 22.9% (249/1804) of the patients have low and high trauma, respectively. In this study, the composition of low and high trauma was not recorded in the registered data. Although that could be surmised according to the injury mechanisms in most of the registered patients, this would result in a limitation in the interpretation of results.

In this study, there was a 1.4-fold and 3.4-fold odds of femoral fracture between high- and medium-risk patients as well as between high- and low-risk patients. Fractures due to osteoporosis represent a serious and costly public health problem and lead to disability and increased mortality of the patients [47]. To reduce these osteoporosis-related fractures, a multidisciplinary approach by the ortho-geriatric model [48], which involving both an orthopedic surgeon and a geriatrician plus a nursing and physiotherapy team, and a fracture liaison service (FLS) [49] had been proposed to reduce re-peat fracture risk and mortality. In addition, the extended assessment will include measures of delirium, prevention of malnutrition, treatment of co-morbidities, and a review of medications with the aim to reduce such medications that promote fall risk or interfere with each other [50]. Some strategies had been implemented to manage the patients with high risk of fracture due to osteoporosis [47], which included 1. At least one session devoted to education to the patients regarding osteoporosis, fracture risk, and medication choices; 2. Adequate calcium, vitamin D, and weight-bearing and resistance exercise; 3. Consider one of some pharmacologic agents to reduce bone resorption and decrease the risk to fracture; 4. Identify and address non-skeletal risk factors for falling and fracture: problems with vision, hearing, balance, home safety adjustments, avoidance of floor rugs, etc. 5. Periodical reassessment in every 1 to 2 years. The patients with a history of prior fracture represent a high risk group and should be targeted for intervention to reduce future fracture rates [51]. For example, the uptake of bisphosphonates and the rollout of public health strategies addressing osteoporosis were suggested to reduce the age-standardized incidence of osteoporotic hip fracture for both females and males in Australia [52].

This study has some other limitations that should be acknowledged. First, in most studies, OSTA demonstrated high sensitivity and low specificity values [17,27,28]. The probability of patients with OSTA score of <−4 having osteoporosis and with an OSTA score of >−4 not having osteoporosis are 53.8% and 99.3%, respectively [23]. This means that a high percentage of subjects categorized as moderate or high risk for osteoporosis by the OSTA actually have normal bone health status based on the bone densitometry (false-positive). Second, some studies showed that OSTA alone did not satisfactorily predict fracture risks in subjects with pre-existing medical conditions [53]. Considering the incidences of comorbidities are higher in high-risk and medium-risk patients than that in the low-risk patients, bias would possibly result among the patients with different risk to osteoporosis. Third, this study focused on hospitalized female patients only; however, some fractures at sites other than the hip are manageable without hospital admissions, which may lead to underestimation on the incidence of these fractures and result in a selection bias. Furthermore, the actual reason of the lower rates of humeral, radial, patellar and tibial fractures high-risk patients than those low-risk patients was unknown. We speculated that is because the femoral bone would share most of the force in the extremities during a fall, thus making a higher incidence of femoral fracture but a reciprocal lower incidence of other fractures in extremities. However, there is lack of evidences in supporting such opinions so far. Finally, an inherent selection bias already existed because of the retrospective study design, particularly when considering the impact force of each injury as well as the drugs for treating osteoporosis was not recorded.

## 5. Conclusions

This study compared the clinical fracture patterns of hospitalized female trauma patients based on the OSTA classification of associated risk to osteoporosis and revealed that the fracture patterns of female trauma patients with high- and medium-risk osteoporosis were different from that of low-risk patients exclusively in the extremity region.

## Figures and Tables

**Table 1:** Demographics and injury characteristics of continuous variables in high-risk (OSTA < −4), medium-risk (−1 ≥ OSTA ≥ −4), and low-risk (OSTA > −1) patients.

Variables	High Risk	Medium Risk	Low Risk	Levene’s Test *p*	*F*	*p*			Mean Difference	Post-Hoc Games-Howell *p*
	−4 > OSTA	−1 ≥ OSTA ≥ −4	OSTA > −1							
	*n* = 1585 (I)	*n* = 1985 (II)	*n* = 3137 (III)							
Age	80.6 ± 6.9	67.9 ± 8.1	55.7 ± 8.7	<0.001	14,518.3	<0.001	Low risk	High risk	−24.9	<0.001
								Medium risk	−12.2	<0.001
Weight	48.6 ± 7.1	56.2 ± 7.6	64.3 ± 10.7	<0.001	8452.6	<0.001	Low risk	High risk	15.7	<0.001
								Medium risk	8.2	<0.001
GCS	14.4 ± 1.9	14.6 ± 1.8	14.6 ± 1.7	<0.001	5.8	0.003	Low risk	High risk	0.2	0.004
								Medium risk	0.0	0.709
ISS	9.7 ± 5.1	8.8 ± 5.8	8.0 ± 6.5	<0.001	40.3	<0.001	Low risk	High risk	−1.6	<0.001
								Medium risk	−0.7	<0.001
LOS	9.6 ± 8.3	8.9 ± 9.1	8.9 ± 9.5	<0.001	3.4	0.032	Low risk	High risk	−0.7	0.206
								Medium risk	−0.0	0.990

GCS = Glasgow Coma Scale; ISS = injury severity score; LOS = length of stay; OSTA = Osteoporosis Self-Assessment Tool for Asians.

**Table 2:** Demographics and injury characteristics of categorical variables in high-risk (OSTA < −4), medium-risk (−1 ≥ OSTA ≥ −4), and low-risk (OSTA > −1) patients.

Variables	High Risk	Medium Risk	Low Risk	OR (95% CI) *p*		OR (95% CI) *p*	
	−4 > OSTA	−1 ≥ OSTA ≥ −4	OSTA > −1	I vs. III		II vs. III	
	*n* = 1585 (I)	*n* = 1985 (II)	*n* = 3137 (III)				
Co-morbidity							
DM	400 (25.2)	580 (29.2)	566 (18.0)	1.5 (1.33–1.77)	<0.001	1.9 (1.64–2.14)	<0.001
HTN	962 (60.7)	974 (49.1)	1002 (31.9)	3.3 (2.90–3.73)	<0.001	2.1 (1.83–2.30)	<0.001
CAD	156 (9.8)	119 (6.0)	84 (2.7)	4.0 (3.02–5.21)	<0.001	2.3 (1.74–3.08)	<0.001
CHF	44 (2.8)	23 (1.2)	21 (0.7)	4.2 (2.51–7.15)	<0.001	1.7 (0.96–3.15)	0.065
CVA	151 (9.5)	116 (5.8)	87 (2.8)	3.7 (2.82–4.84)	<0.001	2.2 (1.64–2.89)	<0.001
Mechanism, *n* (%)							
Motor vehicle	9 (0.6)	16 (0.8)	70 (2.2)	0.3 (0.13–0.50)	<0.001	0.4 (0.21–0.62)	<0.001
Motorcycle	133 (8.4)	712 (35.9)	1659 (52.9)	0.1 (0.07–0.10)	<0.001	0.5 (0.44–0.56)	<0.001
Bicycle	74 (4.7)	103 (5.2)	102 (3.3)	1.5 (1.07–1.98)	0.015	1.6 (1.23–2.15)	0.001
Pedestrian	56 (3.5)	60 (3.0)	70 (2.2)	1.6 (1.12–2.29)	0.009	1.4 (0.96–1.94)	0.079
Fall	1277 (80.6)	1012 (51.0)	960 (30.6)	9.4 (8.13–10.88)	<0.001	2.4 (2.10–2.65)	<0.001
Penetrating injury	7 (0.4)	24 (1.2)	78 (2.5)	0.2 (0.08–0.38)	<0.001	0.5 (0.30–0.76)	0.001
Struck by/against	29 (1.8)	58 (2.9)	198 (6.3)	0.3 (0.19–0.41)	<0.001	0.4 (0.33–0.60)	<0.001
GCS							
≤8	49 (3.1)	54 (2.7)	82 (2.6)	1.2 (0.83–1.70)	0.345	1.0 (0.74–1.48)	0.817
9–12	63 (4.0)	53 (2.7)	73 (2.3)	1.7 (1.23–2.45)	0.001	1.2 (0.81–1.65)	0.440
≥13	1473 (92.9)	1878 (94.6)	2982 (95.1)	0.7 (0.53–0.88)	0.003	0.9 (0.71–1.18)	0.477
AIS ≥ 3, *n* (%)							
Head/Neck	270 (17.0)	273 (13.8)	428 (13.6)	1.3 (1.10–1.53)	0.002	1.0 (0.86–1.19)	0.912
Face	0 (0.0)	2 (0.1)	3 (0.1)	-	0.555	1.1 (0.18–6.31)	1.000
Thorax	34 (2.1)	100 (5.0)	190 (6.1)	0.3 (0.24–0.49)	<0.001	0.8 (0.64–1.06)	0.124
Abdomen	15 (0.9)	25 (1.3)	63 (2.0)	0.5 (0.27–0.82)	0.007	0.6 (0.39–0.99)	0.045
Extremity	991 (62.5)	858 (43.2)	867 (27.6)	4.4 (3.84–4.97)	<0.001	2.0 (1.77–2.24)	<0.001
ISS							
<16	1369 (86.4)	1756 (88.5)	2788 (88.9)	0.8 (0.66–0.95)	0.012	1.0 (0.80–1.15)	0.650
16–24	161 (10.2)	163 (8.2)	251 (8.0)	1.3 (1.06–1.60)	0.013	1.0 (0.84–1.26)	0.788
≥25	55 (3.5)	66 (3.3)	98 (3.1)	1.1 (0.80–1.56)	0.526	1.1 (0.78–1.47)	0.691
Mortality, *n* (%)	44 (2.8)	35 (1.8)	25 (0.8)	3.6 (2.17–5.83)	<0.001	2.2 (1.33–3.74)	0.002
ICU admission, *n* (%)	308 (19.4)	259 (13.0)	386 (12.3)	1.7 (1.46–2.03)	<0.001	1.1 (0.90–1.27)	0.435

AIS = abbreviated injury scale; CAD = coronary artery disease; CHF = Congestive Heart Failure; CI = confidence interval; CVA = cerebral vascular accident; DM = diabetes mellitus; HTN = hypertension; ICU = intensive care unit; OR = odds ratio; OSTA = Osteoporosis Self-Assessment Tool for Asians.

**Table 3:** Associated fracture sites in high-risk (OSTA < −4), medium-risk (−1 ≥ OSTA ≥ −4), and low-risk (OSTA > −1) patients.

Variables	High Risk	Medium Risk	Low Risk	OR (95% CI) *p*		OR (95% CI) *p*	
	−4 > OSTA	−1 ≥ OSTA ≥ −4	OSTA > −1	I vs. III		II vs. III	
	*n* = 1585 (I)	*n* = 1985 (II)	*n* = 3137 (III)				
Head & Face, *n* (%)							
Cranial fracture	34 (2.1)	36 (1.8)	86 (2.7)	0.8 (0.52–1.16)	0.219	0.7 (0.44–0.97)	0.034
Facial bone fracture	28 (1.8)	91 (4.6)	200 (6.4)	0.3 (0.18–0.39)	<0.001	0.7 (0.55–0.91)	0.007
Vertebrae, *n* (%)							
Vertebral fracture	49 (3.1)	82 (4.1)	125 (4.0)	0.8 (0.55–1.08)	0.124	1.0 (0.78–1.38)	0.796
Extremity, *n* (%)							
Humeral fracture	88 (5.6)	159 (8.0)	284 (9.1)	0.6 (0.46–0.76)	<0.001	0.9 (0.71–1.07)	0.196
Radial fracture	169 (10.7)	348 (17.5)	517 (16.5)	0.6 (0.50–0.73)	<0.001	1.1 (0.93–1.25)	0.328
Pelvic fracture	25 (1.6)	54 (2.7)	85 (2.7)	0.6 (0.37–0.90)	0.015	1.0 (0.71–1.42)	0.981
Femoral fracture	829 (52.3)	543 (27.4)	368 (11.7)	8.3 (7.13–9.56)	<0.001	2.8 (2.45–3.28)	<0.001
Patella fracture	25 (1.6)	63 (3.2)	101 (3.2)	0.5 (0.31–0.75)	0.001	1.0 (0.72–1.36)	0.928
Tibia fracture	52 (3.3)	144 (7.3)	298 (9.5)	0.3 (0.24–0.44)	<0.001	0.7 (0.61–0.92)	0.005

CI = confidence interval; OR = Odds ratio; OSTA = Osteoporosis Self-Assessment Tool for Asians.

**Table 4:** Associated fracture sites of propensity-score matched populations with high-risk (OSTA < −4), medium-risk (−1 ≥ OSTA ≥ −4), and low-risk (OSTA > −1) to osteoporosis.

Variables	Propensity-Score Matched Cohort 1				Propensity-Score Matched Cohort 2			
	High Risk	Low Risk	OR (95% CI)	*p*	Medium Risk	Low Risk	OR (95% CI)	*p*
	−4 > OSTA	OSTA > −1			−1 ≥ OSTA ≥ −4	OSTA > −1		
	*n* = 1268 (I)	*n* = 1268 (III)			*n* = 1932 (II)	*n* = 1932 (III)		
Head & Face, *n* (%)								
Cranial fracture	32 (2.5)	23 (1.8)	1.7 (0.92–3.03)	0.089	36 (1.9)	50 (2.6)	0.9 (0.64–1.25)	0.500
Facial bone fracture	25 (2.0)	38 (3.0)	0.7 (0.39–1.09)	0.097	91 (4.7)	100 (5.2)	0.9 (0.68–1.21)	0.504
Vertebrae, *n* (%)								
Vertebral fracture	37 (2.9)	52 (4.1)	0.7 (0.46–1.08)	0.106	81 (4.2)	81 (4.2)	1.0 (0.73–1.37)	1.000
Extremity, *n* (%)								
Humeral fracture	70 (5.5)	121 (9.5)	0.7 (0.50–1.00)	0.047	155 (8.0)	184 (9.5)	1.0 (0.82–1.15)	0.707
Radial fracture	131 (10.3)	257 (20.3)	0.5 (0.39–0.71)	<0.001	334 (17.3)	349 (18.1)	1.0 (0.86–1.14)	0.922
Pelvic fracture	21 (1.7)	20 (1.6)	1.5 (0.69–3.14)	0.324	54 (2.8)	42 (2.2)	1.2 (0.95–1.65)	0.118
Femoral fracture	638 (50.3)	255 (20.1)	3.4 (2.73–4.24)	<0.001	525 (27.2)	302 (15.6)	1.4 (1.24–1.54)	<0.001
Patella fracture	22 (1.7)	76 (6.0)	0.5 (0.27–0.78)	0.004	59 (3.1)	84 (4.3)	0.9 (0.69–1.16)	0.394
Tibia fracture	49 (3.9)	107 (8.4)	0.6 (0.38–0.83)	0.004	142 (7.3)	164 (8.5)	1.0 (0.83–1.18)	0.894

**Table 5:** Demographics of continuous variables in high-risk (OSTA < −4), medium-risk (−1 ≥ OSTA ≥ −4), and low-risk (OSTA > −1) patients in fall accidents.

Variables	High Risk	Medium Risk	Low Risk	Levene’s Test	*F*	*p*			Mean Difference	Post-Hoc
	−4 > OSTA	−1 ≥ OSTA ≥ −4	OSTA > −1	*p*						Games-Howell *p*
	*n* = 1251 (I)	*n* = 996 (II)	*n* = 947 (III)							
Age	81.7 ± 6.6	70.5 ± 8.0	59.2 ± 9.1	<0.001	8606.7	<0.001	Low risk	High risk	−22.5	<0.001
								Medium risk	−11.3	<0.001
Weight	48.8 ± 7.3	58.1 ± 7.8	66.2 ± 10.2	<0.001	4315.6	<0.001	Low risk	High risk	17.4	<0.001
								Medium risk	8.1	<0.001
Height	151.8 ± 5.7	154.6 ± 5.3	157.5 ± 5.3	0.029	291.0	<0.001	Low risk	High risk	5.7	<0.001
								Medium risk	2.9	<0.001

OSTA = Osteoporosis Self-Assessment Tool for Asians.

**Table 6:** Associated extremity fractures in high-risk (OSTA < −4), medium-risk (−1 ≥ OSTA ≥ −4), and low-risk (OSTA > −1) patients in fall accidents.

Variables	High Risk	Medium Risk	Low Risk	OR (95% CI) *p*		OR (95% CI) *p*	
	−4 > OSTA	−1 ≥ OSTA ≥ −4	OSTA > −1	I vs. III		II vs. III	
	*n* = 1251 (I)	*n* = 996 (II)	*n* = 947 (III)				
BMI classification							
Underweight	232 (18.5)	31 (3.1)	8 (0.8)	26.7 (13.13–54.37)	<0.001	3.8 (1.72–8.25)	<0.001
Normal	887 (70.9)	575 (57.7)	351 (37.1)	4.1 (3.46–4.95)	<0.001	2.3 (1.93–2.78)	<0.001
Overweight	130 (10.4)	345 (34.6)	394 (41.6)	0.2 (0.13–0.20)	<0.001	0.7 (0.62–0.89)	0.002
Obese	2 (0.2)	45 (4.5)	194 (20.5)	0.0 (0.00–0.03)	<0.001	0.2 (0.13–0.26)	<0.001
Extremity, *n* (%)							
Humeral fracture	63 (5.0)	75 (7.5)	85 (9.0)	0.5 (0.38–0.75)	<0.001	0.8 (0.60–1.14)	0.247
Radial fracture	134 (10.7)	203 (20.4)	205 (21.6)	0.4 (0.34–0.55)	<0.001	0.9 (0.75–1.15)	0.494
Pelvic fracture	16 (1.3)	16 (1.6)	12 (1.3)	1.0 (0.48–2.14)	0.980	1.3 (0.60–2.70)	0.531
Femoral fracture	750 (60.0)	410 (41.2)	223 (23.5)	4.9 (4.03–5.87)	<0.001	2.3 (1.87–2.76)	<0.001
Patella fracture	22 (1.8)	51 (5.1)	71 (7.5)	0.2 (0.14–0.36)	<0.001	0.7 (0.46–0.97)	0.031
Tibia fracture	16 (1.3)	21 (2.1)	54 (5.7)	0.2 (0.12–0.38)	<0.001	0.4 (0.21–0.59)	<0.001

BMI = body mass index; CI = confidence interval; OR = odds ratio; OSTA = Osteoporosis Self-Assessment Tool for Asians.

## Supplementary Materials

The following are available online at www.mdpi.com/1660-4601/14/11/1380/s1, Table S1: Covariates of high-risk (OSTA < −4) and low-risk (OSTA > −1) patients before and after propensity score matching (1:1 matching via Greedy method), Table S2: Covariates of medium-risk (−1 ≥ OSTA ≥ −4) and low-risk (OSTA > −1) patients before and after propensity score matching (1:1 matching via Greedy method).

## Abbreviations

The following abbreviations are used in this manuscript:

AIS	abbreviated injury scale
BMI	body mass index
BMD	bone mineral density
CAD	coronary artery disease
CHF	congestive heart failure
CIs	confidence intervals
CVA	cerebrovascular accident
DM	diabetes mellitus
ED	emergency department
GCS	Glasgow coma scale
HTN	hypertension
ICU	intensive care unit
IQR	interquartile range
ISS	injury severity score
LOS	length of stay
ORs	Odds ratios
OSTA	Osteoporosis Self-Assessment Tool for Asians

## References

[1] Edwards M.H., Dennison E.M., Aihie Sayer A., Fielding R., Cooper C. (2015). Osteoporosis and sarcopenia in older age. Bone.

[2] National Clinical Guideline (2012). National Institute for Health and Clinical Excellence: Guidance. Osteoporosis: Fragility Fracture Risk: Osteoporosis: Assessing the Risk of Fragility Fracture.

[3] Kanis J.A., Oden A., Johnell O., Jonsson B., de Laet C., Dawson A. (2001). The burden of osteoporotic fractures: A method for setting intervention thresholds. Osteoporos. Int..

[4] Marshall D., Johnell O., Wedel H. (1996). Meta-analysis of how well measures of bone mineral density predict occurrence of osteoporotic fractures. BMJ.

[5] Johnell O., Kanis J. (2005). Epidemiology of osteoporotic fractures. Osteoporos. Int..

[6] Yoo J.H., Moon S.H., Ha Y.C., Lee D.Y., Gong H.S., Park S.Y., Yang K.H. (2015). Osteoporotic Fracture: 2015 Position Statement of the Korean Society for Bone and Mineral Research. J. Bone Metab..

[7] Sanders K.M., Pasco J.A., Ugoni A.M., Nicholson G.C., Seeman E., Martin T.J., Skoric B., Panahi S., Kotowicz M.A. (1998). The exclusion of high trauma fractures may underestimate the prevalence of bone fragility fractures in the community: The Geelong Osteoporosis Study. J. Bone Miner. Res..

[8] Nakaoka D., Sugimoto T., Kaji H., Kanzawa M., Yano S., Yamauchi M., Sugishita T., Chihara K. (2001). Determinants of bone mineral density and spinal fracture risk in postmenopausal Japanese women. Osteoporos. Int..

[9] Watt J., Crilly R. (2017). Location of Vertebral Fractures is Associated with Bone Mineral Density and History of Traumatic Injury. Calcif. Tissue Int..

[10] Kanis J.A., Harvey N.C., Cooper C., Johansson H., Oden A., McCloskey E.V. (2016). A systematic review of intervention thresholds based on FRAX: A report prepared for the National Osteoporosis Guideline Group and the International Osteoporosis Foundation. Arch. Osteoporos..

[11] Reeve J. (2017). Role of cortical bone in hip fracture. Bonekey Rep..

[12] Comim F.V., Marchesan L.Q., Copes R.M., de Vieira A.R., Moresco R.N., Compston J.E., Premaor M.O. (2016). Increased risk of humerus and lower leg fractures in postmenopausal women with self-reported premenopausal hirsutism and/or oligomenorrhea. Eur. J. Obstet. Gynecol. Reprod. Biol..

[13] Bahrs C., Stojicevic T., Blumenstock G., Brorson S., Badke A., Stockle U., Rolauffs B., Freude T. (2014). Trends in epidemiology and patho-anatomical pattern of proximal humeral fractures. Int. Orthop..

[14] Prior J.C., Langsetmo L., Lentle B.C., Berger C., Goltzman D., Kovacs C.S., Kaiser S.M., Adachi J.D., Papaioannou A., Anastassiades T. (2015). Ten-year incident osteoporosis-related fractures in the population-based Canadian Multicentre Osteoporosis Study–comparing site and age-specific risks in women and men. Bone.

[15] Caffarelli C., Alessi C., Nuti R., Gonnelli S. (2014). Divergent effects of obesity on fragility fractures. Clin. Interv. Aging.

[16] Lupsa B.C., Insogna K. (2015). Bone Health and Osteoporosis. Endocrinol. Metab. Clin. N. Am..

[17] Koh L.K., Sedrine W.B., Torralba T.P., Kung A., Fujiwara S., Chan S.P., Huang Q.R., Rajatanavin R., Tsai K.S., Park H.M. (2001). A simple tool to identify asian women at increased risk of osteoporosis. Osteoporos. Int..

[18] Yang Y., Wang B., Fei Q., Meng Q., Li D., Tang H., Li J., Su N. (2013). Validation of an osteoporosis self-assessment tool to identify primary osteoporosis and new osteoporotic vertebral fractures in postmenopausal Chinese women in Beijing. BMC Musculoskelet. Disord..

[19] Machado P., Coutinho M., da Silva J.A. (2010). Selecting men for bone densitometry: Performance of osteoporosis risk assessment tools in Portuguese men. Osteoporos. Int..

[20] Huang J.Y., Song W.Z., Zeng H.R., Huang M., Wen Q.F. (2015). Performance of the Osteoporosis Self-Assessment Tool for Asians (OSTA) in Screening Osteoporosis Among Middle-Aged and Old Women in the Chengdu Region of China. J Clin. Densitom..

[21] Chen C.C., Rau C.S., Wu S.C., Kuo P.J., Chen Y.C., Hsieh H.Y., Hsieh C.H. (2016). Association of Osteoporosis Self-Assessment Tool for Asians (OSTA) Score with Clinical Presentation and Expenditure in Hospitalized Trauma Patients with Femoral Fractures. Int. J. Environ. Res. Public Health.

[22] Rau C.S., Kuo P.J., Wu S.C., Chen Y.C., Hsieh H.Y., Hsieh C.H. (2016). Association between the Osteoporosis Self-Assessment Tool for Asians Score and Mortality in Patients with Isolated Moderate and Severe Traumatic Brain Injury: A Propensity Score-Matched Analysis. Int. J. Environ. Res. Public Health.

[23] Muslim D., Mohd E., Sallehudin A., Tengku Muzaffar T., Ezane A. (2012). Performance of Osteoporosis Self-assessment Tool for Asian (OSTA) for Primary Osteoporosis in Post-menopausal Malay Women. Malays. Orthop. J..

[24] Yang N.P., Lin T., Wang C.S., Chou P. (2004). Correlation of osteoporosis screening by quantitative ultrasound of calcaneus and Osteoporosis Self-Assessment Tool for Asians in Taiwanese. J. Formos. Med. Assoc..

[25] Park H.M., Sedrine W.B., Reginster J.Y., Ross P.D. (2003). Korean experience with the OSTA risk index for osteoporosis: A validation study. J. Clin. Densitom..

[26] Chan S.P., Teo C.C., Ng S.A., Goh N., Tan C., Deurenberg-Yap M. (2006). Validation of various osteoporosis risk indices in elderly Chinese females in Singapore. Osteoporos. Int..

[27] Geater S., Leelawattana R., Geater A. (2004). Validation of the OSTA index for discriminating between high and low probability of femoral neck and lumbar spine osteoporosis among Thai postmenopausal women. J. Med. Assoc. Thai..

[28] Li-Yu J.T., Llamado L.J., Torralba T.P. (2005). Validation of OSTA among Filipinos. Osteoporos. Int..

[29] Bhat K.A., Kakaji M., Awasthi A., Kumar K., Mishra K., Shukla M., Gupta S.K. (2017). Utility of Osteoporosis Self-Assessment Tool as a Screening Tool for Predicting Osteoporosis in Indian Men. J. Clin. Densitom..

[30] Chaovisitsaree S., Namwongprom S.N., Morakote N., Suntornlimsiri N., Piyamongkol W. (2007). Comparison of osteoporosis self assessment tool for Asian (OSTA) and standard assessment in Menopause Clinic, Chiang Mai. J. Med. Assoc. Thail..

[31] Lu C., Chen D., Cai Y., Wei S. (2006). Concordane of OSTA and lumbar spine BMD by DXA in identifying risk of osteoporosis. J. Orthop. Surg. Res..

[32] Hsieh C.H., Hsu S.Y., Hsieh H.Y., Chen Y.C. (2017). Differences between the sexes in motorcycle-related injuries and fatalities at a Taiwanese level I trauma center. Biomed. J..

[33] Hsieh C.H., Liu H.T., Hsu S.Y., Hsieh H.Y., Chen Y.C. (2017). Motorcycle-related hospitalizations of the elderly. Biomed. J..

[34] World Health Organization (1995). Physical Status: The Use and Interpretation of Anthropometry.

[35] World Health Organization (2000). Obesity: Preventing and Managing the Global Epidemic.

[36] Teasdale G., Jennett B. (1974). Assessment of coma and impaired consciousness. A practical scale. Lancet.

[37] Singh B., Murad M.H., Prokop L.J., Erwin P.J., Wang Z., Mommer S.K., Mascarenhas S.S., Parsaik A.K. (2013). Meta-analysis of Glasgow coma scale and simplified motor score in predicting traumatic brain injury outcomes. Brain Inj..

[38] Salottolo K., Levy A.S., Slone D.S., Mains C.W., Bar-Or D. (2014). The effect of age on Glasgow Coma Scale score in patients with traumatic brain injury. JAMA Surg..

[39] Teasdale G., Murray G., Parker L., Jennett B. (1979). Adding up the Glasgow Coma Score. Acta Neurochir. Suppl..

[40] (1971). Rating the severity of tissue damage. I. The abbreviated scale. JAMA.

[41] Baker S.P., O’Neill B., Haddon W., Long W.B. (1974). The injury severity score: A method for describing patients with multiple injuries and evaluating emergency care. J. Trauma.

[42] Keyak J.H., Skinner H.B., Fleming J.A. (2001). Effect of force direction on femoral fracture load for two types of loading conditions. J. Orthop. Res..

[43] Bonnaire F., Zenker H., Lill C., Weber A.T., Linke B. (2005). Treatment strategies for proximal femur fractures in osteoporotic patients. Osteoporos. Int..

[44] Sukumar D., Schlussel Y., Riedt C.S., Gordon C., Stahl T., Shapses S.A. (2011). Obesity alters cortical and trabecular bone density and geometry in women. Osteoporos. Int..

[45] Ebinger T., Koehler D.M., Dolan L.A., McDonald K., Shah A.S. (2016). Obesity Increases Complexity of Distal Radius Fracture in Fall From Standing Height. J. Orthop. Trauma.

[46] Compston J.E., Watts N.B., Chapurlat R., Cooper C., Boonen S., Greenspan S., Pfeilschifter J., Silverman S., Diez-Perez A., Lindsay R. (2011). Obesity is not protective against fracture in postmenopausal women: GLOW. Am. J. Med..

[47] Watts N.B., Manson J.E. (2017). Osteoporosis and Fracture Risk Evaluation and Management: Shared Decision Making in Clinical Practice. JAMA.

[48] Grigoryan K.V., Javedan H., Rudolph J.L. (2014). Orthogeriatric care models and outcomes in hip fracture patients: A systematic review and meta-analysis. J. Orthop. Trauma.

[49] Nakayama A., Major G., Holliday E., Attia J., Bogduk N. (2016). Evidence of effectiveness of a fracture liaison service to reduce the re-fracture rate. Osteoporos. Int..

[50] Pape H.C., Bischoff-Ferrari H.A. (2017). How can we influence the incidence of secondary fragility fractures? A review on current approaches. Injury.

[51] Cummings S.R.N., Nevitt M.C., Browner W.S., Stone K., Fox K.M., Ensrud K.E., Cauley J., Black D., Vogt T.M. (1995). Risk factors for hip fracture in white women. N. Engl. J. Med..

[52] Crisp A., Dixon T., Jones G., Cumming R.G., Laslett L.L., Bhatia K., Webster A., Ebeling P.R. (2012). Declining incidence of osteoporotic hip fracture in Australia. Arch. Osteoporos..

[53] Liu Z., Gao H., Bai X., Zhao L., Li Y., Wang B. (2017). Evaluation of Singh Index and Osteoporosis Self-Assessment Tool for Asians as risk assessment tools of hip fracture in patients with type 2 diabetes mellitus. J. Orthop. Surg. Res..

