# Supplementary material for: Epidemiology of Bone Fracture in Female Trauma Patients Based on Risks of Osteoporosis Assessed using the Osteoporosis Self-Assessment Tool for Asians Score
Source: Int J Environ Res Public Health. 2017 Nov 13;14(11):1380. doi: 10.3390/ijerph14111380 (PMC5708019; doi:10.3390/ijerph14111380)
Supplement: Supplementary file 1 [file ijerph-14-01380-s001.pdf]

Supplemental Tables

Table S1. Covariates of high-risk (OSTA < −4) and low-risk (OSTA > −1) patients before and after propensity score matching (1:1 matching via Greedy method).

Variables	Before matching					After matching				
	**High risk -4>OSTA n=1585 (I)**	**Low risk OSTA>-1 n=3137 (III)**	*OR (95%CI)*	*P*	SD	**High risk -4>OSTA n=1268 (I)**	**Low risk OSTA>-1 n=1268 (III)**	*OR (95%CI)*	*P*	SD
**Mechanism, n (%)**										
**Motor vehicle**	9 (0.6)	70 (2.2)	0.3 (0.13-0.50)	<0.001	–14.20%	9 (0.7)	9 (0.7)	0.4 (0.21-0.62)	<0.001	0.00%
**Motorcycle**	133 (8.4)	1659 (52.9)	0.1 (0.07-0.10)	<0.001	–110.20%	133 (10.5)	133 (10.5)	0.5 (0.44-0.56)	<0.001	0.00%
**Bicycle**	74 (4.7)	102 (3.3)	1.5 (1.07-1.98)	0.015	7.27%	74 (5.8)	74 (5.8)	1.6 (1.23-2.15)	0.001	0.00%
**Pedestrian**	56 (3.5)	70 (2.2)	1.6 (1.12-2.29)	0.009	7.79%	56 (4.4)	56 (4.4)	1.4 (0.96-1.94)	0.079	0.00%
**Fall**	1277 (80.6)	960 (30.6)	9.4 (8.13-10.88)	<0.001	116.33%	960 (75.7)	960 (75.7)	2.4 (2.10-2.65)	<0.001	0.00%
**Penetrating injury**	7 (0.4)	78 (2.5)	0.2 (0.08-0.38)	<0.001	–17.09%	7 (0.6)	7 (0.6)	0.5 (0.30-0.76)	0.001	0.00%
**Struck by/against**	29 (1.8)	198 (6.3)	0.3 (0.19-0.41)	<0.001	–22.83%	29 (2.3)	29 (2.3)	0.4 (0.33-0.60)	<0.001	0.00%

CI = confidence interval; OR = Odds ratio; OSTA = Osteoporosis Self-Assessment Tool for Asians; SD = Standardized Difference.

Table S2. Covariates of medium-risk (−1 ≥ OSTA ≥ −4) and low-risk (OSTA > −1) patients before and after propensity score matching (1:1 matching via Greedy method).

Variables	Before matching					After matching				
	**High risk -4>OSTA n=1585 (I)**	**Low risk OSTA>-1 n=3137 (III)**	*OR (95%CI)*	*P*	SD	**High risk -4>OSTA n=1268 (I)**	**Low risk OSTA>-1 n=1268 (III)**	*OR (95%CI)*	*P*	SD
**Mechanism, n (%)**										
**Motor vehicle**	16 (0.8)	70 (2.2)	0.4 (0.21-0.62)	<0.001	–11.67%	16 (0.8)	16 (0.8)	0.4 (0.21-0.62)	<0.001	0.00%
**Motorcycle**	712 (35.9)	1659 (52.9)	0.5 (0.44-0.56)	<0.001	–34.76%	712 (36.9)	712 (36.9)	0.5 (0.44-0.56)	<0.001	0.00%
**Bicycle**	103 (5.2)	102 (3.3)	1.6 (1.23-2.15)	0.001	9.65%	102 (5.3)	102 (5.3)	1.6 (1.23-2.15)	0.001	0.00%
**Pedestrian**	60 (3.0)	70 (2.2)	1.4 (0.96-1.94)	0.079	4.95%	60 (3.1)	60 (3.1)	1.4 (0.96-1.94)	0.079	0.00%
**Fall**	1012 (51.0)	960 (30.6)	2.4 (2.10-2.65)	<0.001	42.39%	960 (49.7)	960 (49.7)	2.4 (2.10-2.65)	<0.001	0.00%
**Penetrating injury**	24 (1.2)	78 (2.5)	0.5 (0.30-0.76)	0.001	–9.50%	7 (0.6)	7 (0.6)	0.5 (0.30-0.76)	0.001	0.00%
**Struck by/against**	58 (2.9)	198 (6.3)	0.4 (0.33-0.60)	<0.001	–16.21%	29 (2.3)	29 (2.3)	0.4 (0.33-0.60)	<0.001	0.00%

CI = confidence interval; OR = Odds ratio; OSTA = Osteoporosis Self-Assessment Tool for Asians; SD = Standardized Difference.
